# Applications of Deep Learning Models in Laparoscopy for Gynecology

**DOI:** 10.3390/medicina61081460

**Published:** 2025-08-14

**Authors:** Fani Gkrozou, Vasileios Bais, Charikleia Skentou, Dimitrios Rafail Kalaitzopoulos, Georgios Grigoriadis, Anastasia Vatopoulou, Minas Paschopoulos, Angelos Daniilidis

**Affiliations:** 1 Department of Obstetrics and Gynecology, Medical School of Ioannina, University General Hospital, 45500 Ioannina, Greece; 2 Cantonal Hospital of Schaffhausen, 8200 Schaffhausen, Switzerland; dimkal1991@windowslive.com; 3 1st University Department in Obstetrics and Gynecology, Papageorgiou General Hospital, School of Medicine, Aristotle University of Thessaloniki, 54643 Thessaloniki, Greece; drgeorgiosgrigoriadis@gmail.com

**Keywords:** laparoscopy, deep learning, convolutional neural network, artificial intelligence

## Abstract

*Background and Objectives*: The use of Artificial Intelligence (AI) in the medical field is rapidly expanding. This review aims to explore and summarize all published research on the development and validation of deep learning (DL) models in gynecologic laparoscopic surgeries. *Materials and Methods*: MEDLINE, IEEE Xplore, and Google scholar were searched for eligible studies published between January 2000 and May 2025. Selected studies developed a DL model using datasets derived from gynecologic laparoscopic procedures. The exclusion criteria included non-gynecologic datasets, non-laparoscopic datasets, non-Convolutional Neural Network (CNN) models, and non-English publications. *Results*: A total of 16 out of 621 studies met our inclusion criteria. The findings were categorized into four main application areas: (i) anatomy classification (*n* = 6), (ii) anatomy segmentation (*n* = 5), (iii) surgical instrument classification and segmentation (*n* = 5), and (iv) surgical action recognition (*n* = 5). *Conclusions:* This review emphasizes the growing role of AI in gynecologic laparoscopy, improving anatomy recognition, instrument tracking, and surgical action analysis. As datasets grow and computational capabilities advance, these technologies are poised to improve intraoperative guidance and standardize surgical training.

## 1. Introduction

Minimally Invasive Surgery (MIS), first developed in the 1980s and widely adopted in the 1990s, is now the standard approach for numerous procedures [1]. Despite the increase in reported procedures, the number of open surgeries performed continues to decline [2]. In gynecology, MIS is now the preferred approach for most benign and specific malignant conditions [3]. It offers numerous advantages, including decreased blood loss, shorter clinical stay, fewer wound infections, and reduced postoperative pain [4]. However, the technical demands of laparoscopic procedures, including limited degrees of freedom in instrument motion and two-dimensional visualization, pose significant challenges, particularly for inexperienced surgeons [5]. Enhancing intraoperative support through advanced technologies may help achieve more consistent surgical performance.

Deep learning (DL) is a subset of machine learning that uses algorithms inspired by the human brain’s structure and function to learn from unsupervised data [6]. Convolutional Neural Networks (CNNs) are a prominent DL method used in computer vision tasks like image classification, object detection, and segmentation. They consist of three main layers: convolutional layers, which extract features using kernels; pooling layers, which reduce the size of feature maps while preserving important features; and fully connected layers, which convert 2D feature maps into a 1D vector and perform classification. CNNs learn through a forward stage, where an input image passes through the layers, and a backward stage, which adjusts weights to minimize error [7].

DL has gained significant traction in the medical field in recent years. DL has been widely used in medical imaging for tasks such as image segmentation, classification, and reconstruction across various modalities like Magnetic Resonance Imaging (MRI), Computed Tomography (CT), Positron Emission Tomography (PET), ultrasound (US), and Optical Coherence Tomography (OCT) [8]. Beyond radiology, CNNs have demonstrated promising applications in multiple other medical domains, including polyp detection during colonoscopy [9], mitosis identification in breast cancer histology images [10], skin lesions classification in dermatology [11], brain tumor classification [12], and electrocardiogram (ECG) analysis [13], among numerous other uses. Laparoscopy offers large, high-resolution medical image datasets, providing a valuable resource for training CNN models.

CNNs have also been applied in endoscopic surgery, with laparoscopic cholecystectomy being the most frequently studied procedure, accounting for 72.73% of cases. Their applications include surgical tool detection and segmentation, anatomical structure recognition, tissue classification, and surgical phase identification. These technologies hold promise for enhancing intraoperative decision-making and improving surgical precision, though further clinical validation is necessary [14].

Despite growing interest, the use of DL in gynecologic laparoscopy remains relatively underexplored. The large volume of high-resolution visual data generated during laparoscopic procedures presents a valuable opportunity for the effective training of CNNs. This review aims to evaluate the current landscape of CNN applications in gynecologic laparoscopic surgery, focusing on tissue classification, segmentation of anatomical structures, and the automated recognition of surgical instruments and procedural phases.

## 2. Materials and Methods

Search strategy

A comprehensive literature search was conducted across MEDLINE, IEEE Xplore, and Google Scholar to identify studies published between January 2000 and May 2025 that examined DL applications in gynecologic laparoscopic surgery. To ensure completeness, the reference lists of all included studies were also manually reviewed to identify additional relevant publications.

Search queries were tailored for each database as follows:

(1) MEDLINE: “Laparoscopy”[Mesh] AND (“Artificial Intelligence”[Mesh] OR “Deep Learning”[Mesh]) AND (“Gynecology”[Mesh] OR “Hysterectomy”[Mesh] OR “Uterine Myomectomy”[Mesh] OR “Endometriosis”[Mesh]).
(2) IEEE Xplore: Laparoscopy AND Deep learning.
(3) Google scholar: intitle: Laparoscopy OR intitle: Laparoscopic AND “Deep Learning” AND (“Gynecology” OR “Hysterectomy” OR “Uterine Myomectomy” OR “Endometriosis”).

Eligibility criteria

Studies were included if they developed and validated a CNN using datasets from gynecologic laparoscopic surgeries and addressed one or more of the following applications: (i) classification or segmentation of anatomical structures, (ii) detection, classification, or segmentation of surgical instruments, and (iii) recognition of surgical actions or procedural steps. The exclusion criteria were as follows: (i) studies using datasets not exclusively derived from gynecologic laparoscopic surgery, (ii) studies using datasets originating from cadaveric or laparoscopic training boxes, (iii) studies employing DL models other than CNNs, (iv) non-English publications, and (v) non-original research such as abstracts, reviews, editorials, and letters to the editor.

**Figure 1:**
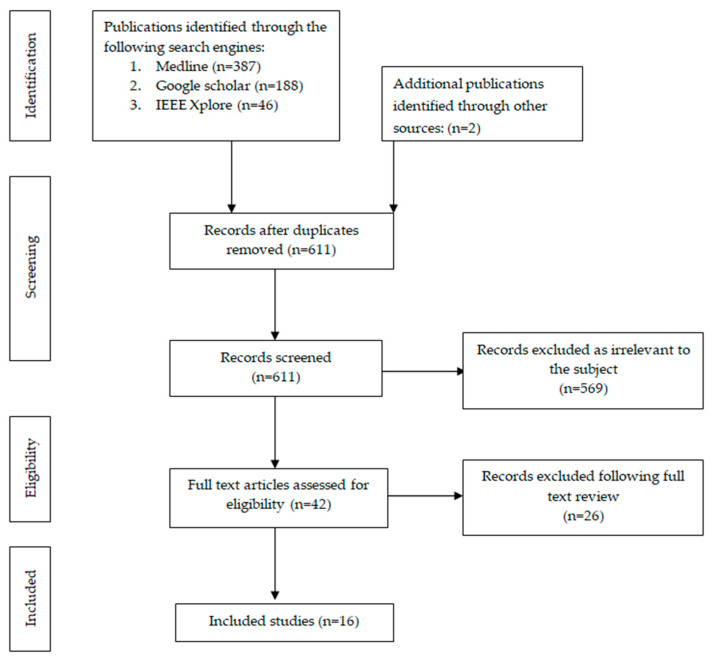
Flowchart of search and inclusion process.

Study eligibility was independently assessed by two reviewers through title and abstract screening. Any disagreements were resolved by a third reviewer. Data extraction was also performed independently and cross-validated for accuracy. The extracted data included the study design, the type of surgical procedures analyzed, the basic CNN architecture employed for model development, the clinical application or task addressed by the model, and the performance metrics reported. A flowchart illustrating the study inclusion process is presented in Figure 1.

Most of the included studies developed CNNs trained on annotated laparoscopic images from gynecologic surgeries, often applying preprocessing techniques such as data augmentation when needed. Models were either trained from scratch with randomly initialized weights or developed through transfer learning by fine-tuning architectures like GoogLeNet Inception v1, developed by Google LLC (Mountain View, CA, USA), that are pretrained on large datasets. CNNs are trained on a training dataset, where they learn to recognize patterns by adjusting their internal parameters. After training, CNNs are evaluated on a separate test set containing new data to measure how well they generalize beyond the training examples. This performance is evaluated by metrics including accuracy, precision, recall, f1 score, Dice score, and intersection over union (IoU) [15].

## 3. Results

The results of the final studies that met our inclusion criteria (*n* = 16) are presented in Table 1. The main findings were as follows: (i) six studies (37.5%) addressed anatomy classification, (ii) five studies (31.2%) focused on anatomical structure segmentation, (iii) five studies (31.2%) covered surgical instrument classification and segmentation, and (iv) five studies (31.2%) investigated surgical action recognition. The evaluation metrics used in this paper are presented in Appendix A Table A1.

### 3.1. Anatomy Classification

Three studies applied CNNs to classify images based on the anatomical structures depicted. Leibetseder et al. implemented GoogleNet for image analysis of their dataset, LapGyn4, which consisted of 111 laparoscopic gynecologic procedures. The model achieved the highest recall rate for the ovary at 94.5%, followed by the colon at 91.9% and the uterus at 91.7%. The oviduct and liver achieved lower recall rates of 84.1% and 82.2%, respectively [20]. Petscharning et al. conducted a similar study, implementing both GoogleNet and AlexNet in their dataset of 111 laparoscopic gynecologic procedures, yielding less impressive results. GoogleNet outperformed AlexNet, achieving recall rates of 88.8% for the ovaries, 86.2% for the liver, 79.5% for the colon, 74.3% for the uterus, and 62.3% for the oviduct [21]. In their recent study, Konduri et al. utilized a full-resolution CNN (FrCNN), which distinguishes itself from traditional CNNs by maintaining the full resolution of input images through the replacement of conventional pooling layers with full resolution layers. LapGyn4 was used as their dataset and achieved outstanding results in organ classification, with an average precision and recall rate of 98.6% and 99.1%, respectively [22].

Three studies [24,25,26] focused on classifying images based on the presence or absence of endometriotic lesions. These studies utilized images from an open-access database, the GLENDA dataset, which comprises a large collection of images from laparoscopic surgery with annotated endometriotic lesions [31]. Among these studies, two [24,25] implemented ResNet50 as the foundational CNN architecture for image analysis and classification, while the third [26] employed eENet. Of note, the study by Nifora et al. demonstrated the highest performance, achieving a precision and recall rate of 99% [24] [Table 2].

### 3.2. Anatomy Segmentation

Three studies applied CNNs to anatomy segmentation [16,17,18]. Madad Zadeh et al. [16] employed Mask R-CNN for image analysis on their proprietary dataset named SurgAI, consisting of eight laparoscopic hysterectomies. The study focused on segmenting the uterus and ovaries; uterus segmentation demonstrated good performance with an IoU of 84.5%, while ovary segmentation performed poorly with an IoU of 29.6% due to limited annotations and significant morphologic variability among patients [16]. In a subsequent study, Madad Zadeh et al. [17] implemented U-Net in their expanded SurgAI3.8K dataset consisting of 79 laparoscopic procedures: 48 hysterectomies, 21 endometriosis excisions, and 10 fertility explorations. The performance of uterus segmentation remained consistent, with an IoU of 84.9% [17].

Serban et al. applied the U-Net architecture to address the challenging task of segmenting critical anatomical structures—uterine arteries, ureters, and nerves—in a proprietary dataset comprising 38 laparoscopic gynecologic surgeries. Accurate segmentation of these structures is clinically significant, as it can help reduce the risk of injury during surgery, where differentiation between these tissues is often difficult. The initial multiclass model demonstrated a limited ability to differentiate between these three structures. To overcome this limitation, the researchers constructed separate binary models for each structure and then combined them using four ensemble techniques. The weighted pixel-wise ensemble technique achieved superior performance in segmenting the ureter, yielding an IoU of 42.01% and a Dice score of 49.99%. However, the weighted region-based ensemble proved more effective in segmenting uterine arteries and nerves, with IoU scores of 27.12% and 55.48%, respectively, along with corresponding Dice scores of 33.72% and 60.25%, respectively [18]. Wang et al. also employed U-Net to segment the ureter in their dataset, which consisted of eleven laparoscopic procedures—two hysterectomies, six hysterectomies with lymphadenectomy, and three endometriosis excisions. After fine-tuning, the model achieved an impressive performance, reaching a Dice coefficient of 77% in an independent test [19].

Leibetseder et al. aimed to advance endometriosis image classification by employing Mask R-CNN and the GLENDA dataset to segment lesions according to their anatomical location, i.e., peritoneum, ovary, uterus, and deep infiltrating endometriosis. They further classified the lesions by visual appearance into categories like ‘mucus’, ‘vesicles’, ‘implants’, and ‘abnormal tissue’. The results for both regional and appearance-based segmentation were disappointing, except for the ‘implants’ category. To address this, they created a new dataset focused solely on ‘implants’ lesions, named ENID, and achieved a mAP50 of 56.1% in segmenting those lesions [27] [Table 3]

### 3.3. Surgical Instruments

Two studies [16,23] focused on surgical instrument segmentation, a third [22] on classifying surgical instruments, a fourth [20] on classifying images by the number of instruments, and a fifth [15] on classifying images based on the presence or absence of instruments. Kletz et al. conducted notable work by applying Mask R-CNN to a proprietary dataset of 333 video frames from laparoscopic hysterectomy and myomectomy procedures. The CNN provided segmentation masks and bounding boxes for eleven surgical instruments: bipolar, grasper, hook, irrigator, knot-pusher, morcellator, needle, needle-holder, scissors, sealer and divider, and trocar. The model achieved an AP50:95 of 42.9%, with an AP50 of 61.3% and an AR of 47.7% [23]. Madad Zadeh et al. utilized Mask R-CNN in the aforementioned SurgAI dataset for surgical tool segmentation without specifying the individual tool, achieving a mean IoU of 54.5%, an AP50 of 88%, and an AR of 86% [16]. Konduri et al. used the LapGyn4 dataset to train the aforementioned FrCNN to classify surgical instruments in the following three categories: grasper, hook, and scissors. The results were highly promising, yielding a precision of 89.6% and a recall of 98.9% [22]. Leibetseder et al. applied GoogleNet to categorize images based on the presence of zero, one, two, or three surgical instruments, with the goal of identifying the corresponding phase of the procedure. The dataset included 411 gynecologic laparoscopic surgeries (LapGyn4) along with the publicly available Cholec80 dataset, which contains images from laparoscopic cholecystectomy procedures. The model demonstrated a strong performance, achieving an average recall of 84.2% and an average precision of 84.1%. Kletz et al., in another study, utilized the LapGyn4 and Cholec80 datasets, employing GoogleNet to classify images as either containing a surgical instrument or not. The CNN was evaluated using a gynecologic dataset, achieving a recall of 95% for instrument presence and 77% for non-instrument presence [15] [Table 4].

### 3.4. Surgical Action Recognition

Leibetseder et al. and Petscharing et al. implemented GoogleNet and both GoogleNet and AlexNet, respectively, in their aforementioned proprietary datasets to classify images based on the surgical actions depicted [20,21]. The surgical actions were categorized into eight types: blunt dissection, coagulation, cutting cold, cutting, hysterectomy (sling), injection, suction and irrigation, and suture. Leibetseder’s CNN achieved a better performance than Petscharing’s, with an average precision rate of 92.4% and a recall rate of 92.5% [20]. In Petscharing’s study, GoogleNet outperformed AlexNet, achieving an average precision of 59% and a recall rate of 61.7% [21]. In a subsequent paper, Petcharing et al. implemented a series of techniques to enhance the input images, yielding less impressive results than Leibetseider et al. [28]. Nasirihaghighi et al. developed a Convolutional Neural Network–Recurrent Neural Network (CNN-RNN) model, where the feature maps from the CNN layers are passed to the RNN layers to capture the temporal dependencies in sequential data. For the CNN backbone, they used VGG16, ResNet50, EfficientNetB2, and DensNeet121, with the RNN component being evaluated using four different architectural types. Their proprietary dataset consisted of 18 laparoscopic gynecologic surgeries. The CNN-RNN model with the best performance utilized ResNet50 as the CNN backbone, achieving an average accuracy of 86.78% for surgical action classification [29]. In addition, Müenzer et al. employed a proprietary dataset called SurgicalActions160, consisting of 160 five-second video clips extracted from gynecologic laparoscopic procedures. They classified these clips using DL methods across 16 surgical actions, ranking them from highest to lowest probability within each category. The average precision for each classification was then calculated, yielding moderate performance results [30]. Leibetseder et al. also trained a CNN to classify images into the following four categories of surgical action on anatomy: suturing the uterus, ovary, vagina, and others. However, the results were less notable, with an average precision of 63.4% and a recall of 62% [20] [Table 5].

## 4. Discussion

This review presents the findings of published studies investigating the application of DL in gynecologic laparoscopic surgeries. The included studies address a range of tasks, including classification and segmentation of both prominent and obscure anatomical structures, surgical instrument detection, and surgical action recognition. Overall, these studies demonstrate progressive advances in applying DL to surgical contexts, evolving from foundational tasks to more advanced applications like specific endometriotic lesion identification and distinct surgical step recognition.

### 4.1. Interpretation of Results

The findings related to anatomical classification and segmentation demonstrate that DL models are effective in identifying key anatomical structures in laparoscopic images derived from gynecologic procedures. Accurate anatomical segmentation through DL models holds significant clinical value by improving intraoperative orientation and enabling safer dissection of pelvic structures. High precision for major organs such as the uterus, ovary, and colon highlights the potential of these models to reduce operative time and prevent complications related to misidentification. This is particularly impactful in complex cases, where identifying key structures can be challenging. However, the performance of DL models remains limited for smaller yet vital structures, such as the ureter and uterine artery, where segmentation errors may directly compromise surgical safety. This is mainly caused by morphological variability and insufficiently annotated datasets. Addressing these gaps is essential to ensure that DL models can support efficiency and safety in surgical practice.

DL models are increasingly capable of correctly identifying surgical instruments, enabling a range of clinically valuable applications. By analyzing tool usage patterns, these models can support the indirect recognition of procedural phases, reducing reliance on manual annotations. Instrument recognition could also aid hospital workflow by linking detected tools to specific procedural codes, supporting accurate billing for consumables. Additionally, real-time detection of inappropriate instrument use near vital structures could serve as a decision support tool, issuing warnings that enhance intraoperative safety. These applications illustrate how DL can move beyond passive recognition toward intelligent surgical assistance systems that promote efficiency and safety in operating room environments.

The recognition of surgical actions and procedural phases by DL models represents a complex yet feasible application. By decomposing complex procedures into clearly defined steps, these models provide a structured framework that helps trainees understand the sequence and purpose of each phase. This enables focused practice of individual surgical phases, allowing learners to develop specific technical skills and strengthen decision-making processes tailored to each step of the procedure. Ultimately, this structured approach leads to better surgical proficiency, increased confidence, and improved competence for real-life operations.

### 4.2. Future Directions

The promising results from DL applications in laparoscopic surgery suggest a bright future, with much potential for further development. A primary future application is the development of real-time DL systems. Most existing models function retrospectively, analyzing images from recorded laparoscopic surgical videos rather than delivering live intraoperative feedback. For clinical utility, DL models must perform within real-time constraints while sustaining high accuracy. Object detection models such as *You Only Look Once* (YOLO), which process images in a single pass with rapid inference speeds, represent promising candidates for real-time surgical applications [32]. Future DL models could provide real-time decision support by recognizing surgical phases, identifying anatomical structures, and flagging potential risks, such as vascular or ureteral proximity during dissection. This could reduce errors and guide less experienced surgeons during complex procedures. Another potential application of DL lies in the objective evaluation of surgical performance. By analyzing tool usage patterns, movement precision, timing, and intraoperative error rates, DL models can generate quantitative assessments of technical skills. In residency training programs, such tools could support individualized feedback, identify specific areas for improvement, and ensure that competency benchmarks are consistently met. Another potential application is the use of DL models in preoperative planning, where analysis of imaging and clinical parameters could help predict surgical complexity and anticipate potential complications. Such predictive capabilities could enhance surgical planning and contribute to more efficient operating room time management. Lastly, by recognizing surgical instruments, procedural phases, and surgeon actions, DL systems have the potential to generate detailed and accurate operative reports in real time. This capability can significantly reduce the bureaucratic workload of surgical teams while maintaining the completeness and quality of surgical documentation.

### 4.3. Limitations

There are several limitations to consider. Firstly, DL models failed to achieve acceptable performance in tasks such as the segmentation of less prominent but clinically crucial structures, such as the ureter, uterine artery, and nerves. This challenge likely arises from the morphological and topographic variability in these structures, as well as from insufficiently annotated or limited datasets. Therefore, the development of large-scale, publicly available, and medically annotated datasets derived from gynecologic surgeries is essential. Existing datasets are often limited to single institutions and lack procedural diversity, which restricts the generalizability and reproducibility of research findings. Expanding the diversity and volume of such data with multi-institutional collaborations will significantly enhance the performance of DL models. Another important limitation is the significant variation in evaluation metrics across studies and applications, making direct comparisons and meta-analyses of data non-feasible. Additionally, most studies are published in computational journals, which often focus on technical aspects rather than medical context, meaning that crucial clinical features and medical insights may be underreported or not sufficiently emphasized. Lastly, although evaluation metrics assess model performance, there is a critical need to conduct large clinical trials to evaluate the impact of DL models on key surgical outcomes, such as operative time, complication rates, and patient recovery, ensuring that these technologies provide measurable benefits to patient care.

## 5. Conclusions

This review highlights the promising results of AI models within the medical field of gynecologic laparoscopy, with applications ranging from foundational to more complex tasks. In the near future, the availability of large, high-quality datasets will support the development of advanced DL models, enabling their application to surgical practice. The successful integration of these models has the potential to significantly impact clinical practices and training protocols, shaping the future of gynecologic surgery. However, the true extent of this impact will need to be evaluated through clinical trials.

## Figures and Tables

**Table 1:** Included studies.

Author	Year	Dataset	Basic Algorithm	Application	Evaluation Metrics
Madad Zadeh S. [16]	2020	SurgAI-Proprietary	Mask R-CNN	(1) Anatomy segmentation (2) Surgical tool segmentation	Mean IoU, Precision ^1^, Recall ^1^
Madad Zadeh S. [17]	2023	SurgAI3.8K-Proprietary	U-Net	Anatomy segmentation	IoU
Serban N. [18]	2024	Proprietary	U-Net	Anatomy segmentation	IoU, Dice score
Wang Z. [19]	2024	Proprietary	U-Net	Anatomy segmentation	Dice score, Hausdorff 95 distance
Leibetseder A. [20]	2018	LapGyn4-Proprietary	GoogleNet	(1) Anatomy classification (2) Instrument count (3) Surgical action and actions on anatomy classification	jacc, rec, prec, spec, mcc, f1
Petscharning S. [21]	2018	Proprietary	GoogleNet, AlexNet	(1) Anatomy classification (2) Surgical action classification	rec, prec, f1, rec@3
Konduri P.S. [22]	2024	LapGyn4	FrCNN	(1) Anatomy classification (2) Surgical instrument classification	acc, pre, rec, f1
Kletz S. [23]	2019	Proprietary	Mask R-CNN	Surgical tool segmentation, bounding boxes	AP 50:95, AP50, AR
Kletz S. [15]	2019	LapGyn4 + Cholec80	GoogleNet	Instrument presence or absence	rec, prec, f1
Nifora C. [24]	2023	GLENDA	ResNet50	Endometriosis or healthy classification	prec, rec, f1, acc
Visalaxi S. [25]	2021	GLENDA	ResnNet50	Endometriosis or healthy classification	prec, rec, f1, acc
Acharya D. [26]	2022	GLENDA	eENet	Endometriosis or healthy classification	prec, rec
Leibetseder A. [27]	2022	GLENDA	Faster R-CNN, Mask R-CNN	Specific endometriosis lesion segmentation	mAP50:95, mAP50, mAP75
Petscharing S. [28]	2018	Proprietary	GoogleNet, AlexNet	Surgical action classification—method comparison	jacc, rec, prec, spec, acc, mcc, f1
Nasirihaghighi S. [29]	2023	Proprietary	CNN-RNN	Surgical action classification	acc
Müenzer B. [30]	2017	SurgicalActions160-Proprietary	HOG, HOF, HMG, AlexNet, GoogleNet	Surgical action classification	prec

IoU: intersection over union; ^1^: precision, recall calculated utilizing IoU threshold of 50%; jacc: Jaccard index; rec: recall; prec: precision; spec: specificity; acc: accuracy; mcc: Matthews correlation coefficient; f1: f1 value; rec@3: recall at 3; AP50:95: precision averaged over IoU thresholds of 50% to 90% obtained in increments of 5%; AP50: average precision for IoU threshold of 50%; AP75: average precision for IoU threshold of 75%; AR: average recall.

**Table 2:** Anatomy classification.

Author	Year	Dataset	Basic Algorithm	Application	Evaluation Metrics
Leibetseder A. [20]	2018	LapGyn4-Proprietary	GoogleNet	Anatomy classification	jacc, rec, prec, spec, acc, mcc, f1
Petscharning S. [21]	2018	Proprietary	GoogleNet, AlexNet	Anatomy classification	rec, prec, f1, rec@3
Konduri P.S. [22]	2024	LapGyn4	FrCNN	Anatomy classification	acc, pre, rec, f1
Nifora C. [24]	2023	GLENDA	ResNet50	Endometriosis or healthy classification	prec, rec, f1, acc
Visalaxi S. [25]	2021	GLENDA	ResnNet50	Endometriosis or healthy classification	prec, rec, f1, acc
Acharya D. [26]	2022	GLENDA	eENet	Endometriosis or healthy classification	prec, rec

jacc: Jaccard index; rec: recall; prec: precision; spec: specificity; acc: accuracy; mcc: Matthews correlation coefficient; f1: f1 value; rec@3: recall at 3.

**Table 3:** Anatomy segmentation.

Author	Year	Dataset	Basic Algorithm	Application	Evaluation Metrics
Madad Zadeh S. [16]	2020	SurgAI-Proprietary	Mask R-CNN	Uterus, ovary segmentation	Mean IoU, Precision ^1^, Recall ^1^
Madad Zadeh S. [17]	2023	SurgAI3.8K-Proprietary	U-Net	Uterus segmentation	IoU
Serban N. [18]	2024	Proprietary	U-Net	Uterine artery, ureter, nerve segmentation	IoU, Dice score
Wang Z. [19]	2024	Proprietary	U-Net	Ureter segmentation	Dice score, Hausdorff 95 distance
Leibetseder A. [27]	2022	GLENDA	Faster R-CNN, Mask R-CNN	Specific endometriosis lesion segmentation	mAP50:95, mAP50, mAP75

IoU: intersection over union; ^1^: precision, recall calculated utilizing the IoU threshold of 50%; AP50:95: precision averaged over IoU thresholds of 50% to 90% obtained in increments of 5%; AP50: average precision for IoU threshold of 50%; AP75: average precision for IoU threshold of 75%.

**Table 4:** Surgical instruments.

Author	Year	Dataset	Basic Algorithm	Application	Evaluation Metrics
Madad Zadeh S. [16]	2020	SurgAI-Proprietary	Mask R-CNN	Surgical tool segmentation	Mean IoU, AP50, AR
Kletz S. [23]	2019	Proprietary	Mask R-CNN	Surgical tool segmentation, bounding boxes	AP50:95, AP50, AR
Konduri P.S. [22]	2024	LapGyn4	FrCNN	Surgical instrument classification	acc, pre, rec, f1
Leibetseder A. [20]	2018	LapGyn4-Proprietary + Cholec80	GoogleNet	Instrument count	jacc, rec, prec, spec, acc, mcc, f1
Kletz S. [15]	2019	LapGyn4 + Cholec80	GoogleNet	Instrument presence or absence	rec, prec, f1

IoU: intersection over union; AP50: average precision for IoU threshold of 50%; AR: average recall; AP50:95: precision averaged over IoU thresholds of 50% to 90% obtained in increments of 5%; jacc: Jaccard index; rec: recall; prec: precision; spec: specificity; acc: accuracy; mcc: Matthews correlation coefficient; f1: f1 value.

**Table 5:** Surgical action recognition.

Author	Year	Dataset	Basic Algorithm	Application	Evaluation Metrics
Leibetseder A. [20]	2018	LapGyn4-Proprietary	GoogleNet	Surgical action and actions on anatomy classification	jacc, rec, prec, spec, acc, mcc, f1
Petscharning S. [21]	2018	Proprietary	GoogleNet, AlexNet	Surgical action classification	rec, prec, f1, rec@3
Petscharing S. [28]	2018	Proprietary	GoogleNet, AlexNet	Surgical action classification—method comparison	jacc, rec, prec, spec, acc, mcc, f1
Nasirihaghighi S. [29]	2023	Proprietary	CNN-RNN	Surgical action classification	acc
Müenzer B. [30]	2017	SurgicalActions160-Proprietary	HOG, HOF, HMG, AlexNet, GoogleNet	Surgical action classification	prec

jacc: Jaccard index; rec: recall; prec: precision; spec: specificity; acc: accuracy; mcc: Matthews correlation coefficient; f1: f1 value; rec@3: recall at 3.

## Data Availability

No new data were created or analyzed in this study. Data sharing is not applicable to this article.

## Appendix A

**Table A1:** Evaluation metrics.

Evaluation Metrics
Accuracy = TP + TN/TP + TN + FP + FN
Precision = TP/TP + FP
Recall = TP/TP + FN
spec = TN/FP + TN
f1 = 2TP/2TP + FP + FN
mcc = TP × TN − FP × FN/$\sqrt{\left(TP+FP\right)\left(TP+FN\right)+(TN+FP)(TN+FN)}$
Jaccard = IoU = |gt ∩ P|/|gt ∪ P|
Dice score = 2|gt ∩ P|/|gt| + |P|
AP50: average precision for IoU threshold of 50%
AP50:95: precision averaged over IoU thresholds of 50% to 90% obtained in increments of 5%
AR: average recall

TP: true positive; TN: true negative; FP: false positive; FN: false negative; IoU: intersection over union; gt: ground truth; P: prediction.
